# Lives Saved Tool supplement detection and treatment of syphilis in pregnancy to reduce syphilis related stillbirths and neonatal mortality

**DOI:** 10.1186/1471-2458-11-S3-S9

**Published:** 2011-04-13

**Authors:** Hannah Blencowe, Simon Cousens, Mary Kamb, Stuart Berman, Joy E Lawn

**Affiliations:** 1 London School of Hygiene and Tropical Medicine, London, UK; 2 Saving Newborn Lives/Save the Children-USA, South Africa; 3 Health Systems Strengthening Unit, Medical Research Council, South Africa; 4 Centers for Disease Control and Prevention, Division of STD Prevention, Atlanta, USA

## Abstract

**Background:**

Globally syphilis is an important yet preventable cause of stillbirth, neonatal mortality and morbidity.

**Objectives:**

This review sought to estimate the effect of detection and treatment of active syphilis in pregnancy with at least 2.4MU benzathine penicillin (or equivalent) on syphilis-related stillbirths and neonatal mortality.

**Methods:**

We conducted a systematic literature review of multiple databases to identify relevant studies. Data were abstracted into standardised tables and the quality of evidence was assessed using adapted GRADE criteria. Where appropriate, meta-analyses were undertaken.

**Results:**

Moderate quality evidence (3 studies) supports a reduction in the incidence of clinical congenital syphilis of 97% (95% c.i 93 – 98%) with detection and treatment of women with active syphilis in pregnancy with at least 2.4MU penicillin. The results of meta-analyses suggest that treatment with penicillin is associated with an 82% reduction in stillbirth (95% c.i. 67 – 90%) (8 studies), a 64% reduction in preterm delivery (95% c.i. 53 – 73%) (7 studies) and an 80% reduction in neonatal deaths (95% c.i. 68 – 87%) (5 studies). Although these effect estimates were large and remarkably consistent across studies, few of the studies adjusted for potential confounding factors and thus the overall quality of the evidence was considered low. However, given these large observed effects and a clear biological mechanism for effectiveness the GRADE recommendation is strong.

**Conclusion:**

Detection and appropriate, timely penicillin treatment is a highly effective intervention to reduce adverse syphilis-related pregnancy outcomes. More research is required to identify the most cost-effective strategies for achieving maximum coverage of screening for all pregnant women, and access to treatment if required.

## Background

Transmission of maternal syphilis infection to the foetus during pregnancy has been described in the medical literature as long ago as 1497 and has been much studied over the past 500 years [1]. However, despite the availability of diagnostic tests for a century and of cheap, effective therapy for over 50 years, WHO currently estimates between 730,000 and 1,500,000 adverse pregnancy outcomes are caused by untreated maternal syphilis each year, of which about 650,000 are foetal and newborn deaths [2,3]. In many regions congenital syphilis remains an important public health problem, e.g Latin America and Africa [2], and the incidence is increasing in some countries, for example China [4,5]. In 2007 WHO launched a programme for the global elimination of congenital syphilis through strengthening existing maternal and newborn health (MNCH) systems in highly affected nations [6].

### Pathophysiology

In 2001, twelve million people globally were estimated to be infected with the causative agent of syphilis, the spirochete *Treponema pallidum* (*T.pallidum*) [7]. Syphilis classically presents in its primary stage with a painless, spontaneously resolving ulcer (chancre), lasting 3 – 6 weeks, that typically occurs around 21 days, but which may occur up to 3 months post infection. This is followed, 6 – 8 weeks later, by the secondary stage, which consists of a more diffuse inflammatory response characterised by an exanthema which manifests variably and for which healthcare is often not sought even if available. This stage also resolves spontaneously and is followed by an asymptomatic ‘latent period’. Untreated, 15 – 40% of infected individuals develop late ‘tertiary’ complications that may occur years after the initial infection: e.g. granulomas, neuropathic joint disease, cardiovascular and neurological complications.

### Diagnosis

Syphilis can be difficult to diagnose, since *T pallidum* cannot be cultured *in vitro*, and PCR-based tests are unavailable outside research settings. While direct diagnosis of characteristic treponemes with darkfield microscopy is highly sensitive and specific, this requires exudate obtained from a lesion during the brief primary stage. Thus most infections are identified using serologic tests that support, rather than establish, a syphilis diagnosis. Serologic tests fall into 2 broad categories: non-treponemal tests (e.g., RPR, VDRL) measure antibody to a component of mammalian cell membranes modified by *T pallidum*. Positive RPR tests are reported as the highest titre for which tests are still positive after dilution, with titre correlating with recentness of infection and stage of disease and falling over time or with treatment. In general, titres >1:4 have been considered to represent “active” infections, more likely to be more recently acquired and infectious. With treatment, titres typically return to negative. Treponemal tests (e.g., TPHA, MHA-TP) detect *T pallidum* antibody, and are reported as either positive or negative. Because these tests typically remain positive even after treatment, a positive treponemal test could indicate either recent active or old (or previously treated) infection. Currently available, rapid point-of-care tests (e.g., ICS strip) are treponemal tests.

### Effects of syphilis on pregnancy outcome

The risk of syphilis to the foetus is dependent upon the stage of maternal infection and on the stage of pregnancy at which the foetus is exposed. Primary, secondary, early latent, and in some cases late latent, stage maternal syphilis infection can lead to haematogenous spread to the foetus, resulting in a systemic inflammatory response [8]. Although spirochetes can cross the placenta to the foetus from early pregnancy [9], foetal immune systems are not mature enough to mount a consistent immune response until about 18 to 22 weeks gestation. After this time the characteristic features of congenital syphilis may be seen. In addition, placental infiltration with reduced blood flow to the foetus can lead to growth restriction which, if severe, may result in foetal death [8]. Foetal involvement occurs most consistently in pregnant women with ‘active’ infections (i.e. RPR titre >1:4), that is inadequately or not treated infections acquired in the five years preceding the pregnancy. This review therefore focuses on this group of pregnant women with active infection. Based on the available data, an expert panel has estimated , that in the absence of effective treatment, 25% of pregnancies to such women will result in a 2^nd^ trimester miscarriage or stillbirth, 11% of pregnancies in a neonatal death at term, 13% in a preterm or low birth weight infant and an additional 20% with clinical signs of congenital syphilis that are attributable to syphilis. Hence, of the 75% of the pregnancies resulting in a liveborn infant, 15% would be expected to result in a neonatal death at term and 17% in a preterm or low birth weight infant, with a further 27% of babies who survive to 28 days developing clinical signs of congenital syphilis [3]. Congenital syphilis in the first 2 years of life may present with hepato-splenomegaly, anaemia, jaundice, rash, snuffles and pseudo-paralysis [10]. Later manifestations of congenital syphilis, after the first 2 years of life may be an important treatable cause of neurological, developmental, and musculoskeletal problems and death in infants and children in low-income settings [11]. In view of the ongoing morbidity and mortality associated with congenital syphilis, and because of the challenges of making a specific diagnosis, both CDC and WHO now recommend a 10 day course of intravenous or intramuscular penicillin treatment for all infants born to mothers with untreated or inadequately treated syphilis in pregnancy , regardless of symptoms or clinical findings [12,13].

### Treatment

Parenteral penicillin was identified as effective in the treatment of syphilis shortly after its introduction in 1943, and is still the recommended drug for treatment of syphilis, whether acquired sexually or through transmission from mother to child during pregnancy [14]. Studies have established that penicillin is treponemocidal at fairly low serum concentrations (>0.1 mug/ml), and that longer duration regimens are more effective than shorter duration regimens, even those achieving higher concentrations [15]. These provide the therapeutic rationale for use of relatively low doses of longer acting penicillin G formulations (e.g. 2.4 million units benzathine penicillin). Current CDC and WHO guidelines recommend a single dose of long acting penicillin to treat early syphilis, and three injections of benzathine penicillin at weekly intervals for infections of > 1 year (or unknown) duration for infected adults, including pregnant women [14]. For women with infection of > 1 year duration (i.e. latent infection), clinical studies suggest that a single penicillin dose effectively treats the foetus in at least 95% of cases [16,17]; while subsequent doses treat the mother. Other antibiotics, such as oral tetracyclines and macrolides (e.g., erythromycin, azithromycin) have been used to treat syphilis in adults, but are not recommended either due to their toxicity in the foetus (tetracyclines) or because they do not cross the placental barrier (macrolides) and thus do not treat foetal infection at all. Macrolide antibiotics have also been associated with development of resistance [15,18]. Parenteral ceftriaxone has been used to treat syphilis in penicillin-allergic patients, but as no controlled studies among pregnant women have assessed its efficacy in treating the foetus, this is not recommended in pregnancy [13,14].

Many factors limit the quality of evidence available concerning the magnitude of the effects of penicillin treatment on foetal and neonatal mortality. Syphilis in pregnancy is a dynamic disease; the timing of infection, re-infection and treatment can all vary, and there are a variety of possible non-specific outcomes (including stillbirth, preterm birth and neonatal and infant death) that are associated with many different, and potentially confounding factors (e.g. other infections, maternal well-being and adherence to recommendations). Given the longstanding recommendations regarding identification and treatment of maternal syphilis randomised controlled trials of the effect of penicillin compared to no treatment would not be ethical. Many observational studies control for potential confounders inadequately or not at all. In summary, despite evidence and widespread policy acceptance of the importance of identifying and treating syphilis during pregnancy, there are limited data regarding the magnitude of the effect such detection and treatment has on foetal and neonatal mortality.

## Objective

The objective of this paper is to provide quantitative estimates of the effect of antenatal syphilis detection based on serological screening in pregnancy combined with treatment with at least 2.4 million units penicillin (at least a single dose of benzathine penicillin or the equivalent multiple dose schedule of shorter acting penicillin) on syphilis related stillbirths, neonatal mortality and morbidity related to maternal syphilis infection. These effect estimates are inputs to the Lives Saved Tool (LiST) [19] and are derived according to set rules [20].

## Methods

We systematically reviewed the published literature to identify studies of detection and treatment of syphilis in pregnancy for the prevention of congenital syphilis mortality (latest searches performed December 2009). There are two broad causal pathways by which active syphilis in pregnancy may lead to mortality in the foetus/ neonate – through direct infection of the foetus and leading to an overwhelming inflammatory response (leading to sepsis, stillbirth or preterm birth) or indirectly through reduced placental blood flow (leading to stillbirth or low birth weight), though this is less frequent. We searched PubMed, EMBASE, Cochrane Libraries, and all World Health Organisation Regional Databases and included publications in any language [20]. Snowball searching was used whereby literature referenced in key papers was included. Combinations of the following search terms were used: *“congenital syphilis/ syphilis” “pregnancy” “neonate/ newborn” “mortality” “screening” “syphilis/ drug therapy” “antibiotics” “preterm” “stillbirth/ foetal death” “perinatal mortality”*.

### Inclusion/ exclusion criteria

**Figure 1:**
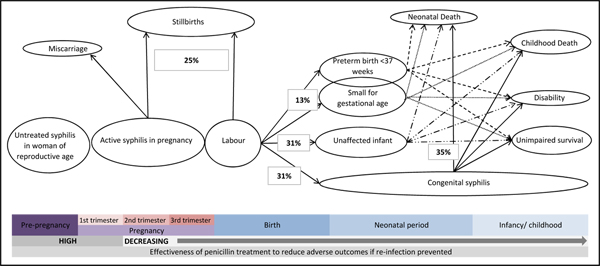
The natural history of untreated syphilis in pregnancy

**Figure 2:**
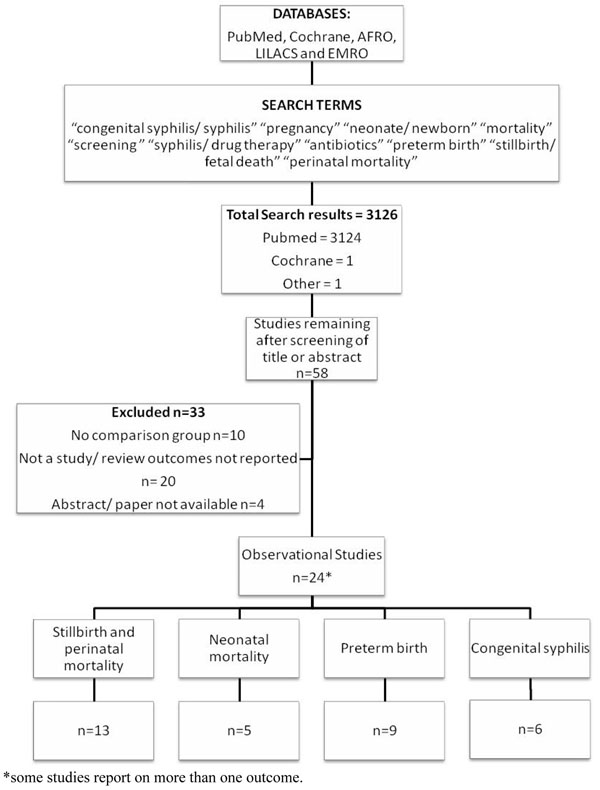
Synthesis of study identification in the review of the effect of at least 2.4MU penicillin on syphilis related adverse pregnancy outcomes

We used the PICO approach (Population, Intervention, Comparison, and Outcome) to identify the studies to be included. The *population* of interest is pregnant women with active syphilis and the *intervention* being reviewed is serologic detection of syphilis in pregnant women and treatment of women with active syphilis (e.g RPR>1:4) with at least 2.4 million units penicillin given at least 28 days prior to delivery. The *comparison* group is pregnant women with active syphilis who do not receive at least 2.4 million units at least 28 days prior to delivery. The *outcomes of interest* are adverse pregnancy outcomes including stillbirth, preterm delivery, congenital syphilis and neonatal mortality associated with congenital syphilis (figure 1). Within this structure we considered both randomised trials and observational studies (figure 2). For duplicate reports of trials or studies we counted outcome data only once, although we have included data from secondary analyses if they added pertinent information. Possible adverse effects of penicillin treatment were not addressed as part of this review.

### Abstraction, analyses and summary measures

All studies meeting the criteria above were abstracted onto a standardised abstraction form for each outcome of interest [20]. Each study was assessed for limitations and graded according to the CHERG adaptation of the GRADE technique [21]. The evidence was summarised by outcome including a qualitative assessment of study quality and sources of bias adapted from the Cochrane review handbook. CHERG Rules for Evidence Review were applied to the collective evidence to provide an estimate for reduction in stillbirth and congenital syphilis related neonatal mortality.

Separate meta-analyses were planned for the effect of penicillin on incidence of stillbirth, preterm delivery, congenital syphilis, and neonatal mortality. Meta-analyses were conducted with STATA version 10.0 statistical software [22]. Heterogeneity was assessed using I^2^ and the chi-squared test. When evidence of heterogeneity was present (p<0.10), a random effects model was used, otherwise a fixed effect was assumed. Summary risk ratios and corresponding 95% confidence intervals (CI) are reported.

## Results

The search strategy from all listed databases and reference lists identified 3126 records (Figure 2). After initial screening of the title or abstract we reviewed 58 papers. Twenty five papers, all observational studies were abstracted and included in the final database. (Additional file 1) Some studies took into account important potential confounders such as co-infection with malaria or anaemia, while others did not. Additionally, some studies assessed pregnancy outcomes in women who were uninfected with syphilis, while others did not. One Cochrane Review (“Antibiotics for syphilis diagnosed during pregnancy”) was retrieved, but included no studies [23].

### 1. Evidence for the effectiveness of detection and treatment of active syphilis in pregnancy in reducing stillbirths and perinatal mortality

Eight observational studies reported the incidence of stillbirths in treated, infected women compared with untreated infected women. None of the studies made any attempt to control this comparison for systematic differences between treated and untreated women (confounding). Women not attending antenatal clinics or not adhering to complex penicillin treatment regimes may differ in their risk profiles for stillbirth, preterm delivery and neonatal death from fully compliant infected women.

Two early, observational studies from the United States report very large reductions in stillbirths to pregnant women identified as ‘syphilitic’ by physical examination and serological testing and treated with at least 2.4 million units of penicillin G compared to untreated syphilis positive women diagnosed at delivery (RRs of 0.15 (95%c.i. 0.10 – 0.25) and 0.06 (95%c.i. 0.01 – 0.27)) [24,25]. Four further studies reported RRs of 0.65 (95% c.i 0.29 – 1.45)[26], 0.17 (0.02 – 1.25) [27] , 0.19 (95% c.i. 0.10 – 0.38)[28] and 0.53 (95%c.i. 0.03 – 9.68)[29] for stillbirth. One study reported no stillbirths in a group of 111 adequately treated RPR-positive women compared to 5 stillbirths in 45 untreated pregnancies RR=0.04 (95%c.i. 0.00 – 0.66) [30]. Two Tanzanian cohorts paired in setting and time [17,31] found three stillbirths amongst 133 women with high titre syphilis who received treatment [17], compared to 18 stillbirths amongst 73 women with high titre syphilis who were untreated[31] RR 0.09 (95% c.i. 0.03 – 0.30).

**Figure 3:**
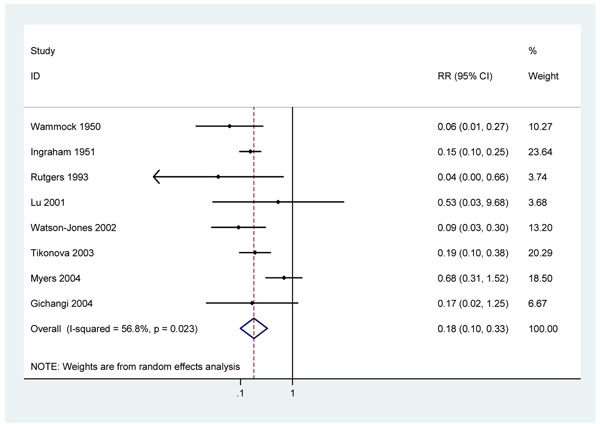
Meta analysis of 8 observational studies showing effect of penicillin on stillbirth in pregnant women with active syphilis

There is some evidence of heterogeneity across these 8 studies (P=0.02; I^2^=56.8%). A random-effects meta-analysis of all eight studies produced an estimated RR of 0.18 (95% c.i. 0.10 - 0.33) (figure 3). The heterogeneity observed appears to be largely attributable to a large South African study [26]. Excluding this study from the analysis there is no longer strong evidence of heterogeneity (P=0.65 I^2^=0%). The summary risk ratio is little changed (RR 0.15: 95% c.i. 0.10 – 0.21 ). The outlying study was relatively large, with a very low number of stillbirths in the untreated group. This may be partly due to some women being recruited in antenatal clinic in the 3^rd^ trimester, and a relatively high loss to follow-up (15%). Since none of these studies controlled for confounding, other factors may have contributed to the observed differences between treated and untreated women.

Five studies compared stillbirth rates in uninfected women compared to infected women who were treated during pregnancy. In three of the studies the observed stillbirth rate were slightly higher among infected women receiving treatment than among uninfected women (RR 1.3; 95% c.i. 0.81 – 2,13[25]: RR 1.4; 95% c.i. 0.34 – 5.75[24]: RR 1.4; 95% c.i. 1.05 – 1.86 [32]) while two studies found no elevated stillbirth risk (RR 0.94: 95% c.i. 0.90 – 0.98 [27]: RR 0.68; 95% c.i. 0.2 – 2.4 [17]).

Four studies investigated the effect of syphilis detection and penicillin treatment on perinatal mortality (i.e. stillbirths and live born infants who died in the first week of life) without distinguishing stillbirths from early neonatal deaths. Two of these studies attempted to control confounding. In one cohort of 158 women with syphilis diagnosed at first ANC visit in rural South Africa, 5/26 untreated pregnancies with known outcomes resulted in perinatal death, compared to 10/116 with at least 2.4 MU penicillin (adjusted RR=0.45: 95%c.i. 0.17 – 1.12) [33]. Another South African study reported a reduction in perinatal mortality of 35% with each dose of penicillin received (adjusted RR = 0.65: 95% c.i. 0.5 – 0.85) [26]. These results are broadly consistent with the two further observational studies that did not control for confounding. A study from South Africa reported a RR of 0.34 (0.11 – 1.03) [34] while a study from Swaziland observed 7/24 perinatal deaths in untreated pregnant women, 0/8 in treated women and 4/145 in those negative for syphilis [35].

### 2. Evidence for the effectiveness of detection and treatment of active syphilis in pregnancy in reducing neonatal mortality

**Figure 4:**
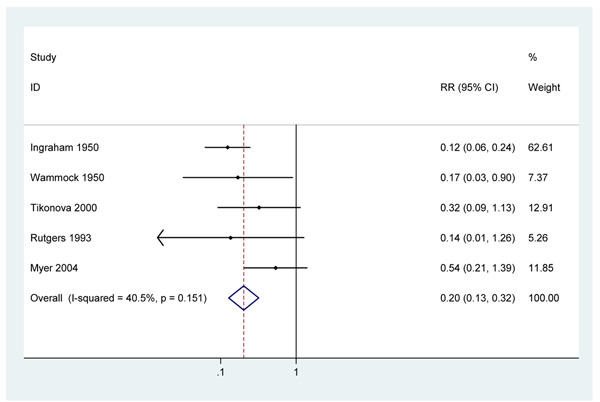
Meta analysis of 5 observational studies showing effect of at least 2.4MU penicillin on all cause neonatal mortality in pregnant women with active syphilis

Two early studies from the United States, report very large reductions in all-cause neonatal mortality in infants of pregnant women with syphilis (RR = 0.14, 95%c.i. 0.07 – 0.27 and RR = 0.18 95%c.i. 0.03 – 0.95) with the use of penicillin therapy [24,25]. The dosing schedules of penicillin administered in these early studies were different to those in current use, using regular dosing with short acting penicillin. Neither study adjusted for potential confounding factors, but both had an additional control group of uninfected women and observed no excess risk of neonatal death in infected women receiving penicillin compared with uninfected women. Three more recent observational studies that also did not adjust for confounding were identified. One study in South Africa recruited women at their first ANC visit and compared those RPR positive women who were treated with penicillin with those who were not treated. To receive treatment women had to return to get their test result. For analysis purposes, only those completing treatment >28 days prior to delivery were considered to have been treated. An all-cause neonatal mortality risk ratio of 0.54 (95%c.i. 0.21 – 1.41) was reported [26]. The treatment effect observed in this study may have been attenuated as screening was based on RPR test positivity without confirmatory testing and will therefore have included some women without active syphilis infection. This is likely to result in an underestimate of the effectiveness of the invention for infected women. The second study, in The Russian Federation, recruited women at delivery, tested them for syphilis using a non-treponemal test for syphilis (Wassermann test) or a treponemal test (fluorescent treponemal antibody absorption) and, if positive, ascertained previous penicillin treatment by examining their treatment cards [36]. This study reported a risk ratio of 0.32 (95% c.i. 0.09 – 1.13) for neonatal death in those treated with penicillin in pregnancy. The third, cross-sectional, study from Zimbabwe used the RPR test to diagnose active syphilis and reported a RR of 0.14 (95% c.i. 0.02 – 1.32) for women treated with 1 dose of benzathine penicillin at least 28 days prior to delivery compared to untreated women [30]. A meta-analysis of these five studies results in an estimated RR of 0.20 (95%c.i. 0.13 – 0.32). (figure 4) There was not strong evidence of heterogeneity (I-squared=40.5%, p=0.15). Given the very large effect size and the biological plausibility of a treatment effect it is unlikely that the differences between treated and untreated women are entirely explained by uncontrolled confounding.

### 3. Evidence for the effectiveness of detection and treatment of active syphilis in pregnancy in reducing preterm births

**Figure 5:**
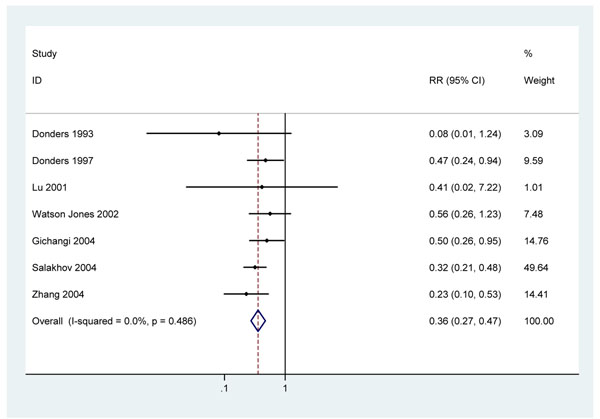
Meta analysis of 7 observational studies showing effect of at least 2.4MU penicillin on preterm delivery in pregnant women with active syphilis

Seven observational studies which did not adjust for confounding were retrieved [24,27,29,34,37-39] and two Tanzanian cohorts paired in time and place [17,31]. One early cohort study from the United States reported a RR 0.18 (95% c.i. 0.03 – 0.95) for preterm birth associated with penicillin treatment but was excluded from further analysis as no definition of preterm birth or how it was assessed was given [24]. The remaining seven studies defined preterm birth as birth before 37 weeks completed gestation and used ultra-sound or last menstrual period and clinical examination at birth to estimate gestational age. A South African cohort reported a RR of 0.47 (0.24 – 0.94) for preterm birth with 1 – 3 doses of 2.4 million units penicillin G compared to no treatment [34]. A second, hospital-based cohort from Kenya which recruited mothers at delivery reported a RR of 0.50 (95% c.i.0.26 – 0.95) for preterm birth with penicillin treatment compared to no treatment [27]. A multi-centre retrospective cohort study in Russia reported a RR for preterm birth of 0.32 (95% c.i. 0.26 – 0.95) comparing adequately treated women to untreated women [38]. One small South African study observed a RR 0.08 (0.01 – 1.24) comparing treated to untreated women [37]. In China one study found a RR 0.23 (0.1 – 0.53) for preterm birth with treatment compared to untreated[39], whilst another small study reported a RR 0.41 (0.02 – 0.72).[29] Re-analysis of the paired Tanzanian cohorts give a RR 0.56 (95% c.i. 0.26 – 1.23). Combining these studies gives a RR of 0.36 (95% c.i. 0.27 – 0.47) for preterm birth with penicillin treatment (figure 5). There was no evidence of heterogeneity p=0.49, I^2^ =0.0%.

Since none of these studies controlled for confounding in this analysis, other factors may have contributed to the association between penicillin treatment and reduced risk of preterm delivery. However the consistency and strength of association and further analyses of the Tanzania cohorts paired in setting and time, which adjusted for potential confounders such as reproductive tract infections, HIV, malaria and anaemia, provide some evidence that the effect seen is not entirely explained by uncontrolled confounding factors. The Tanzania study reported the risk of preterm birth to be 6.1 (95% c.i. 2.5 – 15.3) times higher in women with high titre RPR positivity (i.e., active syphilis) compared to uninfected women[31], while women with high titre RPR positivity who were treated with penicillin had no increased risk of preterm birth (RR =0.58; 95% c.i. 0.3 – 1.1) [17].

### 4. Evidence for the effectiveness of detection and treatment of active syphilis in pregnancy in reducing the incidence of congenital syphilis in live born infants

Three studies were identified addressing the effect of penicillin for pregnant women with active syphilis on the incidence of congenital syphilis. Two early cohort studies in the United States report risk ratios of 0.05 (95% c.i. 0.01 – 0.16) and 0.05 (95% c.i. 0.03 – 0.09) for congenital syphilis [24,25].

A large, population-based study in 6 districts in China screened 477,656 pregnant women using Toluidine Red Unheated Serum Test, a non-treponemal serological test similar to RPR, for detecting current infection [40,41]. Among 2208 (0.5%) test-positive women, the risk of congenital syphilis was 0.004 (7/1855) in those treated with at least 2.4MU IM benzathine benzylpenicillin, and 0.23 (81/353) in those not receiving treatment (RR = 0.02 (95% c.i. 0.01 – 0.04)) [4].

**Figure 6:**
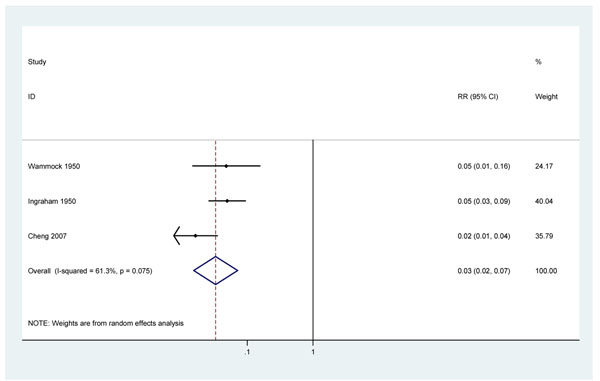
Meta analysis showing moderate evidence of the effect of at least 2.4MU penicillin treatment on incidence of congenital syphilis in pregnant women with active syphilis

These three studies showed some evidence of heterogeneity (p=0.08 I^2^ =61.3%) although all three studies indicate very large effects of treatment with penicillin. This heterogeneity may be partly explained by differences in diagnosis of congenital syphilis between the studies. A random effects meta-analysis produced an estimated risk ratio for congenital syphilis of 0.03 (95%c.i. 0.02 – 0.07) in those women with syphilis in pregnancy who were treated with penicillin, compared to those untreated. (Figure 6)

These results are consistent with a small prospective uncontrolled series examining 11 VDRL positive women identified between fourteen and nineteen weeks gestation. Amniocentesis confirmed foetal infection in four out of 11 cases. All four mothers were treated with 2 doses of 2.4 million units Penicillin G 1 week apart. No infant had serological evidence of congenital infection [9]. Another larger case series of penicillin-treated antenatal attendees with active syphilis reported that 98.2% (95% c.i 96.2 – 99.3%) of pregnancies resulted in an uninfected infant [42]. Timing of treatment is very important in subsequent risk of congenital syphilis. In a large prospective cohort study in China, women commencing treatment before 20 weeks gestation had a lower rate of congenital syphilis RR=0.50 (95%c.i. 0.38 – 0.64) [43] than women treated after 20 weeks.

### Quality of the evidence for estimation of the effect of at least 2.4MU penicillin treatment in pregnant women with active syphilis

The CHERG Rules for Evidence Review were applied [20] (table 1)

**Table 1:** Quality assessment of evidence for treatment with at least 2.4MU penicillin for women with active syphilis in pregnancy to prevent adverse pregnancy and neonatal outcomes:

	Quality Assessment						Summary of Findings					
					**Directness**			**Treated women with active syphilis**		**Untreated women with active syphilis**		

**No of studies (ref)**	**Intervention**	**Design**	**Limitations**	**Consistency**	**Generalizability to population of interest**	**Generalizability to intervention of interest**	**GRADE of evidence**	**Number events**	**Number of births**	**Number events**	**Number of births**	**Effect size (95% CI)**

***Stillbirths:***												

8	At least 2.4MU penicillin	Observational studies	no or insufficient controlling for important potential confounding variables	Consistent	Yes	Yes	Low	70	2578	185	1353	RR= 0.18* (0.10 - 0.33)

***PerinatalMortality (stillbirth and early neonatal mortality):***												

2	At least 2.4MU penicillin	Cohort studies	Both from South Africa	Consistent	Both from South Africa	Yes	Low	39	952	19	233	RR= 0.49 - 0.65

***Neonatal Mortality (All Cause):***												

5	At least 2.4MU penicillin	Observational studies	no or insufficient controlling for important potential confounding variables	Consistent	Yes	Yes	Low	29	2068	62	972	RR=0.20* (0.13 - 0.32)

***Preterm birth:***												

7	At least 2.4MU penicillin	Observational studies	no or insufficient controlling for important potential confounding variables	Consistent	Yes	Yes	Low	85	947	250	1012	RR=0.36* (0.27 - 0.47)

***Congenital syphilis:***												

3	At least 2.4MU penicillin	Observational studies	no or insufficient controlling for important potential confounding variables but as very specific outcome unlikely to be confounded	Consistent	Yes	Yes	Moderate	20	2745	139	715	RR=0.03* (0.02 - 0.07)

*Based on calculated pooled estimate

#### 1. Evidence of effect on perinatal deaths and stillbirths

Combining data from eight cohort studies that did not control for potential confounding results in an estimate for the reduction in stillbirth amongst women with active syphilis in pregnancy of 82% (95% c.i. 67 – 90%) with penicillin treatment (figure 3). There is a very large, biologically plausible and consistent effect size. Since none of these studies adequately controlled for potential confounding factors, other factors may have contributed to this apparent relationship, although the strength and the consistency of the association makes it rather unlikely that that the association can be entirely explained by confounding factors. Also, several studies included a comparison group of uninfected women and noted similar stillbirth rates in this group to the rates in syphilis-infected women who were treated, providing further evidence that the observed reductions in stillbirths were due to treatment of syphilis. However, without adjustment for confounding factors the quality of evidence is considered low.

Four studies which examined perinatal mortality were all consistent with a substantial reduction with penicillin treatment.

#### 2. Cause-specific evidence of effect on congenital syphilis on deaths in the neonatal period

There is no high or moderate quality direct evidence for the effect of treating women with syphilis in pregnancy on reduction of congenital syphilis specific-neonatal deaths. In five observational studies, treatment was consistently associated with a reduction all-cause neonatal mortality of 80% (95% c.i. 68 – 87%) (figure 4). Some studies included a comparison group of uninfected women and noted that neonatal mortality in syphilis-infected women who were treated was similar. This provides some circumstantial evidence that the observed reductions in neonatal mortality may have been due to treatment of syphilis. None of the studies controlled for possible confounding factors and hence the quality of the evidence is low.

#### 3. Cause-specific evidence of effect on preterm birth

Seven observational cohort studies which did not control for potential confounders in this analysis consistently reported reductions in preterm births with penicillin of 64% (95% c.i. 53 – 73%) (figure 5). The overall quality of evidence is low and distal to the mortality effect. Some studies included a comparison group of uninfected women with birth outcomes similar to infected women treated with penicillin. This provides some supportive evidence that the observed reductions in preterm birth may have been due to treatment of syphilis. The quality of the evidence is low.

#### 4. Evidence of effect on incidence of congenital syphilis

Combining data from 3 observational studies results in an estimated reduction in the incidence of clinical congenital syphilis of 97% (95% c.i. 93 – 98%) with penicillin (figure 6). There is a very large, biologically plausible and broadly consistent effect size which, given the very specific outcome, is unlikely to be confounded by other factors, so GRADE rules result in upgrading the quality of evidence from low to moderate.

#### Estimation of effect on neonatal mortality

A recent expert consensus panel reviewing published data has estimated that approximately 15% of livebirths in women with active syphilis in pregnancy result in a neonatal death from congenital syphilis and that a further 27% have clinical evidence of syphilis in the first year of life [3]. Current practice, based on both the WHO and CDC guidelines for the management of presumptive congenital syphilis, includes parenteral penicillin treatment for all infants born to untreated or inadequately treated mothers[12,13]. Whilst this will markedly reduce the incidence and later morbidity and mortality of congenital syphilis it is likely to have less impact on neonatal mortality as most of the neonatal deaths from congenital syphilis occur on the first day of life [44,45].

In a high prevalence setting in Malawi in 1990, with a “very high” neonatal mortality NMR typology of 47/1000, about 30% (14 per 1000) of all neonatal deaths would be expected to be due to sepsis [46]. With a prevalence of active syphilis of 3.6%, without treatment in pregnancy, for every 1000 pregnant women 36 women would be infected with active syphilis of whom 9 (25%) would be expected to have a stillbirth and 27 a livebirth. Overall 4 women (11% of pregnancies, 15% of livebirths) would expect to result in a term neonatal death attributable to syphilis i.e. a “sepsis” death. Assuming 97% efficacy about 8% (3.88/47) of overall neonatal deaths and 28% (3.88/14) of neonatal sepsis deaths in women with syphilis could be averted.

This is likely to be a conservative estimate. Additional neonatal deaths attributable to maternal syphilis are expected due to the effect of syphilis in pregnancy on preterm delivery and low birth weight, with their associated increased neonatal mortality. Four recent studies report 17% (90/536) of live births in women with syphilis in pregnancy were preterm [27,31,47,48]. Preterm delivery was reported six times more frequently in RPR positive women compared with RPR negative women [31].

## Discussion

Mortality from untreated maternal syphilis remains an important yet preventable cause of neonatal deaths and stillbirths. The systematic review identified five relevant studies, suggesting a large and consistent effect size on all-cause neonatal mortality for women with untreated syphilis in pregnancy but none controlled for confounding. Unsurprisingly, no randomised trials have examined the effect of penicillin or alternative treatments for syphilis in pregnancy which would have provided high quality direct evidence regarding the neonatal cause-specific mortality effect of penicillin treatment for women with syphilis in pregnancy. There is low quality evidence of the effect of penicillin in reducing all cause perinatal mortality. Based on studies of the incidence of congenital syphilis (3 studies, 159 cases,) we estimate that prenatal detection of maternal syphilis infection and treatment with at least 2.4 million units of penicillin reduces the incidence of congenital syphilis by 97% (95% c.i 93 – 98%). Assuming conservatively that penicillin treatment in pregnancy does not affect the case fatality from congenital syphilis, the reduction in congenital syphilis neonatal mortality will be equal to the reduction in incidence i.e. 97%. Assuming 15% of livebirths in women with active syphilis in pregnancy result in a congenital syphilis-related neonatal death at term, treatment before the last trimester of pregnancy with at least 2.4MU penicillin would be expected to avert 97% of these deaths. (Table 2) The effect on neonatal mortality is likely to be underestimated as the six-fold increase in preterm birth is not taken into account in this estimate. In addition, this review has, of necessity, been based on studies in which treatment was given at least 28 days prior to delivery. However, studies support the belief that treatment earlier in pregnancy ideally before 24 – 28 weeks gestation has a greater effect on pregnancy outcome and hence we may be underestimating the effect of earlier treatment. Thirdly this does not take into account late foetal demise or stillbirth, the most common pregnancy outcome caused by untreated maternal syphilis, estimated to occur in about 25% of affected pregnancies, or of later infant deaths from congenital syphilis.

**Table 2:** Cause specific mortality effect and quality grade of the estimate for the effect of at least 2.4MU penicillin treatment for active syphilis in pregnancy


***Cause specific mortality to act on:***
The proportion of stillbirth mortality that is related to syphilis.
The proportion of preterm mortality that is related to syphilis.
The proportion of neonatal sepsis mortality that is due to congenital syphilis.

***Cause specific estimate of effect in affected pregnancies and range:***
Reduction in stillbirth of 82% (95% c.i. 67 – 90%)
Reduction in the incidence of preterm delivery of 64% (95% c.i. 53 – 73%)
Reduction in the incidence of clinical congenital syphilis of 97% (95% c.i. 93 – 98%)
Assuming 15% of livebirths from syphilis affected pregnancies result in a neonatal death attributable to congenital syphilis (expert consensus) and that treatment does not affect case fatality, 97% (95%c.i. 93 – 98%) of these deaths could be averted.

***Quality of input evidence:***
For stillbirth – There is a large and consistent effect size from 8 observational studies. The GRADE quality of evidence is low.
For preterm - There is a large and consistent effect size from 7 observational studies. The GRADE quality of evidence is low.
There is a large and consistent effect size on incidence of clinical congenital syphilis and confounding is unlikely, so GRADE rules result in upgrading the low to moderate quality evidence.
The GRADE recommendation is STRONG, based on large and consistent effect sizes across studies, and biological plausibility.

***Proximity of the data to cause specific mortality effect:***
For the effect on preterm and neonatal sepsis due to congenital syphilis, the evidence is on incidence and distal to mortality estimate.

***Possible adverse effects:***
Penicillin allergic symptoms may occur in 2% of individuals [89]; however, severe allergic reactions (e.g. anaphylaxis) are rare events, estimated to occur in 10 to 50 of every 100,000 exposed individuals [90,91]. Up to 40% of patients with primary or secondary syphilis may experience Jarisch-Herxheimer reactions with antibiotic treatment. These reactions involving malaise, anxiety, fever, headache, sweating, or rigors are believed caused by microbial lysis. If the reaction occurs late pregnancy it could lead to fetal distress, uterine contractions and preterm delivery [92,93]. There is some evidence of possible increased maternal infection after antepartum treatment [94]


The information used on the extent to which maternal syphilis infection affects pregnancy outcome is based on a variety of sources. Randomised controlled trials of the effect of penicillin compared to no treatment would not be ethical. Studies reporting on the effect of syphilis in pregnancy on pregnancy outcome e.g. stillbirth, neonatal death, infected infant most commonly compare the outcome of untreated pregnancies with treated pregnancies or outcome of infected pregnancies versus non-affected pregnancies. In both cases the two groups may differ with respect to other risk factors for neonatal outcome, potential confounders (e.g. maternal age), co-infections (e.g. malaria and HIV) and maternal anaemia. Two recent studies which sought to control for potential confounding variables, have reported on the association of untreated active syphilis in pregnancy (RPR/VDRL titre >= 1:4 and reactive MHA-TP assay) with peri- and neonatal outcomes in low income settings. A study from Malawi, in a population with a prevalence of active syphilis of 3.6%, reported an adjusted odds ratio (OR) of 10.89 (95% c.i 6.61 – 17.93) for stillbirth and 4.86 (95% c.i. 2.73 – 10.36) for neonatal death [49]. An estimated 11% (95% c.i. 6 – 17%) of neonatal deaths and 26% (95% c.i.18 – 35%) of stillbirths in this population were attributable to syphilis. A cohort study in Tanzania in which 7.7% of women tested seropositive for syphilis, reported adjusted risk ratios (RR) of 18.1 (95% c.i.5.5 – 59.6) for stillbirth, of 3.3 (95% c.i. 2.0 – 5.4) for low birth weight (LBW), of 6.1 (95% c.i. 2.5 – 15.3) for preterm birth, and of 2.1 (95% c.i. 1.0 – 4.2) for intra-uterine growth retardation (IUGR) [31].

A limitation of the GRADE approach, well illustrated in the present context, is its potential to produce results which could be misinterpreted by the reader who glances at the results but does not have time to read them and the conclusions carefully. There can be no doubt, based on our understanding of biological mechanisms, and the consistent and large effects seen across multiple studies, that timely diagnosis and treatment with benzathine penicillin of active syphilis infection in pregnant women substantially reduces the risk of stillbirth, and neonatal morbidity and mortality. However, all of the available quantitative evidence regarding the precise magnitude of these reductions comes from observational studies. Such evidence is automatically classified as "low quality" by the GRADE process unless one can essentially rule out any confounding (or other threats to validity). One is very rarely in a position to do this. The GRADE approach is, however, a two stage process, first grading the quality of evidence and secondarily the strength of recommendation. This is an intervention which should clearly be strongly recommended (GRADE recommendation STRONG) in areas where syphilis is prevalent even though it comes with a tag of "low quality evidence" attached. We should also emphasise the distinction between the quality of the evidence assigned by the GRADE process and the quality of individual studies contributing to that evidence. In many cases, well designed and executed observational studies will produce only “low quality evidence” in GRADE terms.

We assessed interventions that involved any treatment with at least 2.4 million units of penicillin, and did not attempt to compare longer versus shorter penicillin regimens. Foetal syphilis infection is, by definition, of less than one year duration, and thus falls under WHO and CDC treatment recommendations for early infection of a single intramuscular dose of 2.4 million units of benzathine penicillin (although effective treatment of a mother with latent infection would require additional doses per those recommendations). Watson-Jones found such a single dose treatment to be effective in preventing syphilis-associated adverse pregnancy outcomes among Tanzanian women attending ANC and, importantly, that 229 women ≤ 28 weeks gestation who received a single dose of penicillin had no increased risk for adverse pregnancy outcome compared with seronegative women[17]. Gestational age at treatment is likely quite important, as foetal inflammatory response to treponemal infection occurs in the latter part of the 2^nd^ trimester of pregnancy. Detection and treatment of syphilis infection later than 24 to 28 weeks gestation may be too late to prevent occurrence of some foetal losses, stillbirths or preterm births. Completing 3 penicillin doses has been observed to be highly correlated with gestational age at diagnosis, with women attending ANC later in pregnancy less likely to receive all 3 doses [50]. While some studies have reported multiple penicillin doses to be more effective than single dose regimens in reducing adverse pregnancy outcomes associated with maternal syphilis infection, these did not report results by gestational age at first treatment [26,34]. It is possible that longer regimens may confer some additional benefit in reducing adverse pregnancy outcomes, particularly among women co-infected with HIV [51] or in women with secondary infection [42]; however we were not able to assess this using existing data.

Detecting and measuring accurately the incidence of congenital syphilis is problematic even in countries with comprehensive surveillance systems [52]. False positives may occur with RPR/ VDRL for example with yaws, viral infections, autoimmune conditions and pregnancy. These tests are user-dependent and onsite testing reduces sensitivity [47,53-55] whilst laboratory-based screening involves women returning for the result. This delay in initiating treatment may result in fewer women receiving treatment or in insufficient time for treatment to be adequately effective prior to delivery. The most practical approach in low income settings is to ensure early screening and prompt, ideally same visit, treatment of positives, with follow-up treatment of mothers with additional penicillin doses if required e.g. late latent or tertiary syphilis. In settings lacking laboratory capacity for RPR testing this may be best achieved through use of point-of-care treponemal tests [56]. This approach is currently being implemented in several countries. A recent cluster randomised trial found using point-of-care treponemal tests and first dose of penicillin in a one-stop clinic reduced congenital syphilis by 93.5% (95% c.i 66.0%–98.6%) compared with off-site RPR testing and penicillin treatment at next ANC visit [57]. However, current point-of-care tests do not distinguish between past and present infection [58] and thus may result in some over-treatment of positive mothers who have been previously treated. Studies suggest that risks to the mother and costs of unnecessary treatment are small [14,59]; however, introduction of a dual treponemal/non-treponemal point of care test, anticipated in the near future, should help address this problem.

Whilst policies for syphilis control are present in over three-quarters of 22 countries surveyed in Sub-Saharan Africa, in practice many women remain unscreened and untreated. Even amongst those receiving treatment, it is often only received after the critical first 24 weeks of pregnancy [60]. In a study in Tanzania, only 43% of women attending ANC were screened and only 61% of those positive and 37% of their partners were treated [61]. Other studies report similar figures [47,62-68]. To prevent syphilis related adverse pregnancy outcomes women must access ANC sufficiently early, be tested, receive results promptly, be treated adequately, and remain uninfected for the rest of pregnancy [69]. Implementing programmes can be difficult and even in high income countries cases of syphilis-related stillbirth and congenital syphilis still occur [70]. Contributing factors include failure to attend ANC or attending only in the third trimester, lack of testing supplies/ transport for specimens and treatment available at the antenatal site, not returning for results/ treatment, infection or re-infection during pregnancy, and (rarely) failure of treatment [70-72]. Resolving some of these operational issues may be easier with decentralised approaches but concerns exist regarding sustainability [73-75].

How to screen most efficiently for syphilis is currently the major issue for low income countries [69]. The economic cost of screening and treating cases of syphilis in pregnancy in Tanzania has been estimated to be $1.44 per women screened, $20 per woman treated and $186 per case of stillbirth or low birth weight averted [76]. Modelling has suggested on-site testing with ICS to be the most effective and least costly strategy [77] and translation of these results into effective routine programmes has been demonstrated [59,75]. Discussion surrounds whether screening should be carried out once or twice during pregnancy, though ensuring screening once for every woman would represent a major step forward. 0.4 – 2.8% of pregnant women in high prevalence areas were found to have become seropositive for syphilis between testing in the 1^st^ and the 3^rd^ trimester [17,78-80]. Contact tracing of partners to reduce the risk of re-infection is resource intensive and rarely carried out [34]. It is unclear what proportion of cases of congenital syphilis attributed to ‘penicillin treatment failure’ are actually due to re-infection [65,80-84].

Co-infection with HIV infection compromises the serological response to and probably effectiveness of syphilis treatment of syphilis in adults [51,85]. The effect of HIV on treatment of syphilis in pregnancy is not known. However syphilis may increase the transmission of congenital HIV infection [86,87]. Introducing a functioning programme of syphilis screening and treatment into already established prenatal HIV testing services may be a cost-effective strategy and an opportunity for cohesion to maximise prevention of mother to child transmission of both HIV and syphilis [59,88].

## Conclusion

Evidence supports that at least 2.4MU penicillin given at least 28 days prior to delivery is effective in the treatment of syphilis in pregnant women to prevent congenital syphilis, with greatest effect when given early in pregnancy (before 24 -28 weeks). Our estimates suggest such treatment results in at least a 97% reduction in the incidence of congenital syphilis. Assuming that penicillin treatment does not alter case fatality from congenital syphilis, 97% of neonatal deaths from congenital syphilis may be averted by appropriate timely penicillin treatment for the affected mother during pregnancy. In addition, low grade evidence consistently supports large reductions amongst infected women in risks of stillbirth (82%), preterm birth (64%) and neonatal mortality (80%) with appropriate and timely treatment but uncontrolled confounding factors may account for part of these apparent effects.

Detection and appropriate timely penicillin treatment is an effective intervention to reduce adverse syphilis-related pregnancy outcomes. The strength of GRADE recommendation is strong based on biological plausibility and consistent and large effects seen across all studies reviewed. More research is required to identify the most cost effective strategy for achieving maximum coverage of screening for all pregnant women and access to treatment if required.

### Funding

This work was supported in part by a grant to the US Fund for UNICEF from the Bill &Melinda Gates Foundation (grant 43386) to “Promote evidence-based decision making in designing maternal, neonatal and child health interventions in low- and middle-income countries”, and by a grant to Save The Children USA from the Bill & Melinda Gates Foundation (Grant 50124) for "Saving Newborn Lives".

## Competing interests

The authors declare that they have no competing interests.

## Authors’ contributions

SC and JL planned the review with HB who undertook the searches and abstraction. HB produced the meta-analysis. HB and JL drafted the manuscript and all authors contributed

## Supplementary Material

Additional file 1 Syphilis Web Table.pdf Click here for file
